# Behavioural state inference from movement and environmental data using Markovian step selection functions

**DOI:** 10.1371/journal.pone.0360547

**Published:** 2026-10-26

**Authors:** Ilhem Bouderbala, Aurélien Nicosia, Daniel Fortin

**Affiliations:** 1 Mathematics Department, King Fahd University of Petroleum and Minerals, Dhahran, Saudi Arabia; 2 Département de biologie, Université Laval, Québec (Qc), Canada; 3 Interdisciplinary Research Center for Biosystems and Machines, King Fahd University of Petroleum and Minerals, Dhahran, Saudi Arabia; 4 Département de Mathématiques et Statistique, Université Laval, Québec (Qc), Canada; 5 Centre interdisciplinaire en modélisation mathématique de l’Université Laval, Québec (Qc), Canada; Tsinghua University, CHINA

## Abstract

Animal movement data often contain multiple behavioural states that vary across space and time, but identifying these states in a biologically interpretable way remains challenging. We propose an ecologically guided framework for inferring individual behavioural states from movement trajectories using hidden Markov models with step selection functions (HMM-SSFs). The method combines a small set of a priori, biologically motivated state hypotheses with explicit ordering and distinctness criteria on movement signatures, together with model-adequacy diagnostics and a stable fitting strategy. Unlike fully unsupervised approaches, our framework is designed to select among a limited number of ecologically meaningful behavioural states rather than exhaustively search a large latent state space. Simulations show that the method can recover the intended state structure under different behavioural scenarios, and that inference is sensitive to the specification of control locations in the SSF component. An illustrative application to a plains zebra trajectory demonstrates how the framework can be used to infer and label behavioural states from movement data. Overall, the proposed workflow provides a practical and reproducible way to infer individual-level behavioural states while maintaining ecological interpretability.

## 1 Introduction

Movement is a fundamental process that influences not only individual fitness but also shapes population dynamics, and community structure and composition [1,2]. Animal movement paths reflect intricate, context-dependent responses to internal states and external environmental conditions. A wide-range of factors, including hunger, predation risk, spatial memory, locomotor capacity, and landscape structure, can influence movement behaviour [3,4]. The relative importance of these drivers often varies across space and time, resulting in movement patterns composed of multiple behavioural states expressed across diverse spatio-temporal scales [5,6]. For example, grazing large herbivores exhibit hierarchical foraging behaviours, moving among feeding stations within vegetation patches and across multiple feeding sites composed of such patches [7,8]. At broader scales, their movements are typically structured around camps, distinct areas within their home range that encompass several feeding sites [5].

The hierarchical nature of resource use, ranging from short-duration visits to feeding stations (<2 min) to prolonged stays at camps (1–4 weeks), implies that movements within and between these levels should exhibit distinct behavioural signatures. Variations in basic movement metrics, such as step length (SL) and turning angle (TA), as well as drivers of directional bias, can differentiate among behavioural states [9,10]. However, as movement data are collected over extended periods, both the number of meaningful behavioural states and the diversity of influencing factors tend to increase [7,11]. This growing complexity poses challenges the objective identification of behavioural states and their underlying drivers, highlighting the need for analytical approaches capable of detecting states in multiphasic movement paths while maintaining both objective and biologically relevance.

Hidden Markov Models (HMMs) provide a robust framework for inferring latent behavioural states from animal movement trajectories [11–14]. HMMs have been integrated with both generalized biased and correlated random Walk models (BCRW) and Step Selection Functions (SSFs) to study the interplay between habitat conditions and multiphasic movement patterns. Compared to BCRW, recent studies often favour SSFs because they can be estimated with widely statistical software and provide a simple tool to evaluate multiple movement drivers simultaneously [15–18]. Despite differences, both (BCRW) and SSFs can effectively distinguish movement states and identify their ecological drivers. Notably, the two approaches have been shown to be functionally equivalent under specific control locations sampling conditions [15,16].

HMMs are widely used to study animal movement and can be trained under various learning paradigms, depending on the availability of information about the underlying behavioural states [19]. In a *supervised* setting, the number of states, their ecological interpretations, and the true state at each time point are all known, typically obtained through direct observation or auxiliary sensor data [20]. This allows direct estimation of transition and state-dependent parameters without decoding the hidden state sequence. In contrast, *unsupervised* HMMs assume no prior knowledge of the number of states, their labels, or their temporal assignments. Instead, the model jointly estimates these quantities from the data, and state labels are assigned post hoc based on ecological interpretation. In such cases, statistical criteria based on information theory or penalised likelihood methods are often used to determine the optimal number of states [21,22]. A *semi-supervised* approach lies between these extremes: part of the state sequence is known, while the remainder is inferred [23]. We additionally distinguish a *constrained* setting, in which the number of states and their ecological labels are specified a priori, but the exact state at each time step remains unknown and must be inferred from the data [10,16,17]. This latter case is particularly relevant when the number of states is fixed a priori to reflect ecologically meaningful behaviours (e.g., travelling vs. encamped). By contrast, fully unsupervised models are entirely data-driven, with the model structure inferred solely from the data and without explicit ecological constraints. Although such approaches can be effective for exploratory analyses, they may yield models with limited ecological interpretability. Importantly, the specification of an a priori behavioural taxonomy (e.g., “travelling” vs. “encamped”) does not guarantee that the fitted states will correspond to these labels; such correspondence must be evaluated using explicit, ecologically motivated criteria applied to the estimated state-dependent movement signatures.

In this article, we adopt an ecological guided workflow, widely used in ecological research, in which model selection is guided by predefined, biologically plausible hypotheses. Our framework relies on an *unsupervised* learning strategy with explicit ecological constraints: a limited set of candidate state numbers and their biological interpretations is defined *a priori*, while the temporal sequence of states is inferred directly from movement data. To prevent arbitrary proliferation of states, candidate models are required to satisfy predefined orderings of movement metrics, ensuring that inferred states correspond to ecologically meaningful patterns. Behavioural states are characterised using SL, angular concentration, directional persistence, and movement biases relative to habitat features, and these criteria are jointly used to infer both the number of states and their biological interpretation. Rather than seeking a unique “true” number of behavioural states, our objective is to select among a small set of ecologically motivated state taxonomies by evaluating their consistency with the data, diagnostic checks, and the expected ordering of state-dependent movement signatures. This strategy preserves ecological interpretability while avoiding both the rigidity of fully supervised models and the overfitting risks of unconstrained unsupervised approaches.

This perspective aligns with the pragmatic view advocated by Pohle et al. [21], who showed that information criteria can be unreliable for HMM order selection and recommended combining candidate-model fitting with careful inspection of state-dependent distributions, decoded sequences, and formal model checking. Our contribution builds directly on this foundation by (i) operationalising ecologically motivated behavioural taxonomies through explicit ordering and distinctness criteria, and (ii) embedding these criteria within a reproducible, algorithmic workflow complemented by model adequacy diagnostics. While similar principles could be implemented within other directional-bias HMM formulations, we present the framework using HMMs with step selection functions (HMM-SSFs), as this formulation naturally accommodates case–control sampling, importance-sampling corrections under non-uniform availability, and direct ecological interpretation of habitat-selection coefficients.

Compared with existing HMM-SSF analyses and standard HMM workflows, our contribution is not a new state-dependent likelihood, but a more explicit model-selection and interpretation framework. Rather than relying only on AIC/BIC or software defaults to choose the number of states, we combine HMM-SSF estimation with a priori ecologically motivated taxonomies, post-fit ordering and distinctness checks on movement signatures, and diagnostic screening to ensure that the retained states are biologically interpretable and numerically stable.

## 2 Materials and methods

### 2.1 Hidden Markov model step selection function (HMM-SSF)

HMM-SSF characterise transitions between movement states (e.g., stationary vs. exploratory) using a hidden Markov process to capture state dependence [9]. For each observed step $\left\{y_{t},y_{t+1}\right\}$, *J* control steps $\left\{Z_{1,t},...,Z_{J,t}\right\}$ are generated, where TA and SL may be sampled uniformly, i.e., TA~𝒰(0,2π) and SL~𝒰(0,M), with *M* denoting the maximum inter-location distance. Alternatively, control points can be drawn from empirical or parametric distributions [17,24,25]. The conditional likelihood of observing a step ending at $y_{t+1}$, given origin $y_{t}$ and latent state $S_{t+1}=k$, is approximated by:


$$\begin{array}{c} Pr\left(y_{t+1}|S_{t+1}=k,y_{t}\right)=\frac{\exp\left(c_{0,t}^{T}.\beta^{\left(k\right)}\right)}{\sum_{l=0}^{J}\exp\left(c_{l,t}^{T}.\beta^{\left(k\right)}\right)} \end{array}$$ (1)

where $c_{l,t}=c\left(y_{t},Z_{l,t}\right)$ is the covariate vector obtained by evaluating the step-covariate function c(·,·) at the pair $\left(y_{t},Z_{l,t}\right)$, i.e., the feature vector describing the step from origin $y_{t}$ to location $Z_{l,t}$, $Z_{0,t}=y_{t+1}$. Here, *l* = 0 indexes the observed step and l=1,...,J index the *J* control steps. $\beta^{\left(k\right)}$ denotes the state-specific parameters, representing the effect of each covariate on step selection. Additionally, transition probabilities may depend on *p* environmental covariates, time of day, and interactions with neighbouring animals, modelled using a multinomial-*l*ogit link function [11,17].


$$\begin{array}{c} \pi_{ij,t}=Pr\left(S_{t+1}=j|S_{t}=i\right)=\frac{\exp\left(\gamma_{ij,t}\right)}{\sum_{k=1}^{K}\exp\left(\gamma_{ik,t}\right)} \end{array}$$

with


$$\begin{array}{c} \left\{ \begin{array}{c} \gamma_{ij,t}=\alpha_{ij,0}+\alpha_{ij,1}x_{1,t}+...+\alpha_{ij,p}x_{p,t}\text{ if }i\neq j\\ 0\text{ if }i=j \end{array}\right. \end{array}$$

where $\left\{x_{1},...,x_{p}\right\}$ denote the environmental covariate vector, $\alpha_{ij,0}$ is the state-pair-specific intercept for the transition from state *i* to state *j*, and $\alpha_{ij,1},...,\alpha_{ij,p}$ are the corresponding regression coefficients quantifying the effect of each covariate on that transition probability.

Klappstein et al. [17] proposed an approach to correct for the influence of non-uniform control sampling or a low number of control points. Their method adjusts the likelihood using an *importance sampling correction*, which accounts for the bias introduced by the control distribution. In this framework, the step likelihood is approximated as:


$$\begin{array}{c} Pr\left(y_{t+1}|S_{t+1}=k,y_{t}\right)=\frac{\exp\left(c_{0,t}^{T}.\beta^{\left(k\right)}\right)}{\sum_{l=0}^{J}\exp\left(c_{l,t}^{T}.\beta^{\left(k\right)}\right)/h\left(y_{t},Z_{l,t}\right)} \end{array}$$ (2)

where $h(y_{t},Z_{l,t})$ denotes the probability density function of the control distribution used to sample control locations. This correction ensures the likelihood properly accounts for deviations from uniform sampling, enhancing the robustness of SSF-based movement models under different control schemes. The modeller can either: (i) maximise Eq 1 and adjust the SL and TA parameters, or (ii) directly maximise the corrected likelihood in Eq 2.

### 2.2 Clustering metrics

Clustering metrics are used to operationalise biological hypotheses about behavioural differentiation and to guide model selection among candidate state-space models. Because behavioural modes vary across species and ecological contexts (Table 1), the choice of metrics must be tailored to the focal ecological question and the hypothesised behavioural taxonomy. For each fitted candidate model with a fixed number of states *K*, we extract a vector of clustering metrics Ψ from the estimated state-dependent movement parameters. These metrics typically include summaries of SLs, TA concentration, directional persistence, or context-specific measures such as movement bias toward known locations or responses to environmental risk. For basic behavioural contrasts (e.g., encamped, resting, foraging, travelling), SLs and angular concentration are often sufficient. For example, Fryxell et al. [6] distinguished encamped from exploratory behaviour using the frequency of directional reversals, while Ylitalo et al. [26] identified homing behaviour through high speeds and directed movement toward a known site. More complex behavioural distinctions may require additional or context-specific metrics (Table 1; [10]).

**Table 1:** Examples of movement states with their description.

Movement state	State description	Reference
Foraging	Low angular concentration, moderate SLs	[6]
Searching	Concentrated TA, relatively long SLs	
Resting	Shelter retreat: low angular concentration, short SLs	[27]
Foraging	Food search: relatively long SLs	
Encamped	Near resource patches: low angular concentration, short SLs	[10]
Exploratory	§Away from resources/predators: concentrated TA, relatively long SLs	
Feeding site	Short SLs	[5]
Camp area	Low angular concentration, intermediate SLs	
Resting	Very short SLs	[28]
Encamped	Low angular concentration, moderate SLs	
Exploratory	High directional persistence, long SLs	
Resting	Low angular concentration, short SLs	[26]
Moderate speed travel	Intermediate angular concentration, intermediate SLs	
Fast travel	Low angular concentration, long SLs	
Homing	Long SLs, movement bias towards the den	

Importantly, specifying an *a priori* behavioural taxonomy (e.g., “travelling” vs. “encamped”) does not guarantee that fitted states will align with those labels. Such alignment must be evaluated using explicit, ecologically motivated criteria applied to the estimated state-dependent movement signatures. In our framework, these criteria are encoded through an ordering logic defined on the clustering metrics Ψ. For each candidate model, we therefore evaluate whether the extracted metric vector Ψ satisfies the user-defined ordering logic that reflects the hypothesised behavioural structure. This logic specifies expected qualitative relationships among states, for instance, increasing SL and TA concentration from resting to foraging to travelling. Model adequacy is assessed by determining whether the estimated metrics exhibit biologically interpretable separation and conform to these expectations. If the ordering logic is violated or yields ambiguous or ecologically implausible state differentiation, the candidate model is rejected and a simpler model with fewer states is fitted. This procedure is repeated iteratively until a model is obtained for which the clustering metrics satisfy the hypothesised behavioural ordering. Once a candidate model passes this validation step, final behavioural labels are assigned to states, and the most probable state sequence is decoded for the full trajectory. In this way, clustering metrics do not define behavioural states directly, but provide an explicit and ecologically grounded criterion for validating model structure and guiding state-number selection.

#### Environmental covariates for ecological state labelling.

Beyond movement-derived metrics, environmental covariates play a central role in assigning ecologically meaningful labels to the inferred behavioural states. In the HMM–SSF framework, such covariates are introduced at the step-selection level and interpreted conditionally on the movement state, thereby complementing the movement-based clustering metrics Ψ.

Environmental covariates can be broadly classified into three categories. First, numerical covariates *h*(*x*) describe continuous habitat attributes measured at the step endpoint or along the step, such as elevation, vegetation density, distance to water, or predation risk. State-specific selection coefficients associated with these covariates quantify attraction or avoidance of environmental gradients and help distinguish behaviours linked to resource exploitation or risk minimisation.

Second, directional covariates capture orientation biases relative to external features through terms of the form $\cos(\theta -\theta^{\text{target}})$, where θ denotes the movement bearing and $\theta^{\text{target}}$ the direction toward a biotic or abiotic target (e.g., a refuge, food patch, den, or conspecific). A significant positive coefficient indicates directed movement toward the target, whereas non-significant effects suggest habitat-independent displacement. These covariates are particularly informative for discriminating habitat-dependent from habitat-independent travelling behaviours.

Third, categorical or indicator covariates encode the presence or absence of specific habitat types or landscape elements (e.g., grassland vs. woodland, inside vs. outside a protected area). In this case, state-specific coefficients measure differential use of discrete habitat classes and can be used to identify behaviours associated with habitat residency, patch use, or transitions between habitat types.

Crucially, ecological state labelling relies on the joint interpretation of movement metrics Ψ and environmental covariates, rather than on either component alone. Movement-derived quantities characterise the intrinsic locomotory mode, while environmental covariates reveal the external drivers shaping movement decisions within each state. This joint perspective ensures that behavioural states are defined not only by how animals move, but also by why and where they move, providing a coherent ecological interpretation of the inferred state structure.

### 2.3 Example taxonomy and conditions

In this section, we define the movement-based component Ψ and illustrate the proposed framework using a simple, ecologically interpretable taxonomy centred on a directional target. This choice reflects a common situation in movement ecology, where behavioural states are distinguished not only by intrinsic movement characteristics, but also by orientation toward a specific biotic or abiotic feature. We derive a set of clustering metrics Ψ:


$$\begin{array}{c} \Psi =(\mu_{SL},\sigma_{SL},\ell ,\beta_{\cos(TA)},\beta_{\cos(\theta -\theta^{\text{target}})}), \end{array}$$

where $\mu_{SL}$ and $\sigma_{SL}$ are the mean and standard deviation of the step-length (SL) distribution for a given state, ℓ is the angular concentration parameter of the turning-angle distribution (a scalar ≥0; larger values indicate more directed movement), θ denotes the animal’s bearing direction and $\theta^{\text{target}}$ represents the direction toward influential biotic or abiotic habitat attributes, such as a predator, prey, or refuge. In this formulation, $\beta_{\cos(TA)}$ is the coefficient associated with the cosine of the TA, and $\beta_{\cos(\theta -\theta^{\text{target}})}$ represents the coefficient associated with the cosine of the angular difference between the step direction θ and the direction toward the influential habitat attribute(s) $\theta^{\text{target}}$.

Together, these clustering metrics provide a comprehensive description of both intrinsic movement properties and environmentally driven directional responses, ensuring that the resulting states are ecologically interpretable. SL is assumed to follow a gamma distribution, and TAs follow a von Mises distribution, two of the most commonly used distributions in animal movement modelling [9,17]. The parameters Ψ are derived from the estimated coefficients of *SL*, log(SL), and cos(TA) obtained from the HMM–SSF model [17], as follows:


$$\begin{array}{c} \left\{ \begin{array}{c} \mu_{SL}=-\frac{\beta_{\log(SL)}+2}{\beta_{SL}},\;\sigma_{SL}=\frac{-\sqrt{\beta_{\log(SL)}+2}}{\beta_{SL}},\\ \ell =|\beta_{\cos(TA)}| \end{array}\right. \end{array}$$ (3)

where $\beta_{SL}$ and $\beta_{\log(SL)}$ are the HMM–SSF regression coefficients associated with the raw step length *SL* and its logarithm log(SL), respectively, from which the gamma-distribution parameters $\mu_{SL}$ and $\sigma_{SL}$ are recovered [17].

The sign and magnitude of $\beta_{\cos(TA)}$ provide direct insight into movement directionality: values of $\beta_{\cos(TA)}\gg 0$ indicate strong directional persistence, whereas $\beta_{\cos(TA)}\ll 0$ indicate a strong tendency to reverse direction [17,18]. These clustering metrics are subsequently used to identify the states that best describe the observed movement data under the sets of conditions described below (Conditions 1–3), each corresponding to state-switching dynamics associated with a specific behavioural label (see Algorithm 1).

**Algorithm 1. Guided model selection and state labelling**

**Require:** Case-control data 𝒟; SSF formula with direction-to-target; TPM formula; $\left(\underset{―}{\pi },\overset{―}{\pi }\right)$

Ensure: Selected number of states $\hat{K}$, clustering metrics $\left\{\Psi_{k}\right\}$, labels, decoded states ${\hat{S}}_{1:T}$

1: **Step 1 — HDT/F/R (3-state with habitat-direction covariate, Condition 1)** {Test whether directed movement toward a habitat target is detectable}

2: Fit HMM(3)-SSF with covariates $\{SL,\log SL,\cos TA,\cos(\theta -\theta^{\text{target}}\}$

3: Decode ${\hat{S}}_{1:T}$ (Viterbi); compute $\Psi_{k}$; check TPM bounds and occupancy

4: **if** Condition 1 satisfied **and** $\beta_{\cos(\theta -\theta^{\text{target}})}^{\left(HDT\right)}$ significant **then**

5:  Assign labels HDT, F, R {Habitat-directed travelling state confirmed}

6:  **Return** $\left(3,\{\Psi_{k}\},\{HDT,F,R\},{\hat{S}}_{1:T}\right)$

7: **else**

8:  {Either ordering violated, TPM/occupancy failed, or habitat-direction}

9:  {covariate not significant ⇒ drop it and retry with movement only}

10: **end if**

11: **Step 2 — T/R/F (3-state without habitat-direction covariate, Condition 2)** {Test whether three states are still distinguishable by movement metrics alone}

12: Refit HMM(3)-SSF with covariates {SL,logSL,cosTA} only

13: Decode ${\hat{S}}_{1:T}$; compute $\Psi_{k}$; check TPM bounds and occupancy

14: **if** Condition 2 satisfied **then**

15:  Assign labels T, R, F {Generic travelling distinguished from resting/foraging}

16:  **Return** $\left(3,\{\Psi_{k}\},\{T,R,F\},{\hat{S}}_{1:T}\right)$

17: **else**

18:  {Ordering violated or two states statistically indistinguishable}

19:  {⇒ collapse to a 2-state model}

20: **end if**

21: **Step 3 — T/E (2-state, Condition 3)** {Minimal meaningful contrast: directed displacement vs. localised activity}

22: Fit HMM(2)-SSF with covariates {SL,logSL,cosTA}

23: Decode ${\hat{S}}_{1:T}$; compute $\Psi_{k}$; check TPM bounds and occupancy

24: **if** Condition 3 satisfied **then**

25:  Assign labels T, E {Two ecologically distinct states retained}

26:  **Return** $\left(2,\{\Psi_{k}\},\{T,E\},{\hat{S}}_{1:T}\right)$

27: **else**

28:  {No multi-state solution satisfies biological criteria}

29:  {⇒ movement path treated as behaviourally homogeneous}

30: **end if**

31: **Fallback — single state** {All multi-state models rejected; report undifferentiated movement}

32: Fit 1-state SSF; compute $\Psi_{1}$; label as “Other”

33: **Return** $\left(1,\Psi_{1},\text{Other},{\hat{S}}_{1:T}\right)$

#### Three-State model (*K*= 3).

We begin by considering a model comprised of three potential behavioural states: resting, foraging, and habitat-dependent travelling (HDT). The animal is assumed to remain mostly immobile when in the resting state, such that $\mu_{SL}$ is relatively small. For simplicity, we consider that the foraging state is comprised of a single movement mode, independent of specific habitat features and without specific expectation for TAs ($\beta_{\cos(TA)}$) [6,9,10,16]. However, it is worth noting that some animals employ foraging strategies, such as area-restricted search, that involve more than a single movement state, differing in movement metrics, and that can be linked to habitat structure and composition [10,29–31]. Studies with sufficiently high-resolution movement data may find it valuable to subdivide the foraging state accordingly.

In the HDT state, movement is influenced by habitat characteristics, which may include both abiotic (e.g., topography, roads) and biotic factors (e.g., competitors, predators). HDT is expected to display directional persistence (large $\mu_{SL}$ and high angular concentration ℓ), such that $\beta_{\cos(TA)}^{\left(HDT\right)}>0$, combined with a significant orientation bias toward the focal habitat features. This state may emerge, for example, when an individual moves toward a refuge or resource patch [32,33]. In such cases, the 3-state label should satisfy all conditions:

- **Habitat-Dependent Travelling, Foraging, and Resting.** This case applies when the direction-to-target covariate is included and $\beta_{\cos(\theta -\theta^{\text{target}})}^{\left(HDT\right)}$ is statistically significant, confirming habitat-driven displacement.The 3-state label should satisfy:

$$\begin{array}{c} \left\{ \begin{array}{c} \mu_{SL}^{\left(HDT\right)}>\mu_{SL}^{\left(F\right)}>\mu_{SL}^{\left(R\right)},\\ \ell^{\left(HDT\right)}>\ell^{\left(F\right)},\\ \beta_{\cos(TA)}^{\left(HDT\right)}\geq 0,\\ \text{significant}\beta_{\cos(\theta -\theta^{\text{target}})}^{\left(HDT\right)}. \end{array}\right. \end{array}$$ (Condition 1)

A second pathway for reducing model complexity arises when the specification is over-parametrised for the available data. We then refit the model and re-assess the behavioural-state conditions. We distinguish (i) conceptual collapsing of states when two hypothesised modes are statistically indistinguishable, from (ii) numerical or data-support limitations (non-convergence, unstable estimates), which trigger a refit with fewer states without an ecological ‘collapse’ interpretation.

- **Travelling, Foraging, and Resting.** This case applies when no environmental predictor is included to define behavioural states, or when $\beta_{\cos(\theta -\theta^{\text{target}})}^{\left(HDT\right)}$ is not significant in Condition 1. The model distinguishes generic Travelling (T), Foraging (F), and Resting (R) via:

$$\begin{array}{c} \left\{ \begin{array}{c} \mu_{SL}^{\left(T\right)}>\mu_{SL}^{\left(F\right)}>\mu_{SL}^{\left(R\right)},\\ \ell^{\left(T\right)}>\ell^{\left(F\right)},\\ \beta_{\cos(TA)}^{\left(T\right)}\geq 0, \end{array}\right. \end{array}$$ (Condition 2)

#### Two-State models (*K* = 2).

We consider in this model two states travelling and encamped. Travelling is characterised by longer SLs and higher directional persistence, whereas encamped behaviour is more variable, typically involving shorter SLs and more frequent reversals. The encamped state can encompass a mix of low-mobility behaviours and can be further subdivided into foraging and resting states [21].


$$\begin{array}{c} \left\{ \begin{array}{c} \mu_{SL}^{\left(T\right)}>\mu_{SL}^{\left(E\right)}\\ \ell^{\left(T\right)}>\ell^{\left(E\right)}\\ \beta_{\cos(TA)}^{\left(T\right)}\geq 0 \end{array}\right. \end{array}$$ (Condition 3)

#### One-state model (*K* = 1).

For *K* = 1, the animal’s behaviour is categorised as a single, undifferentiated state designated as “Other.”

### 2.4 How to define behavioural conditions in practice

In practice, behavioural conditions should be defined from the combination of the ecological question, the temporal resolution of the data, the available covariates, and prior knowledge about the study system. The ecological question determines which contrasts are meaningful for a given species or system, such as travelling versus encamped movement, foraging versus resting, or habitat-dependent versus habitat-independent movement. The temporal resolution then determines which of these contrasts can realistically be resolved: fine-scale telemetry may support separation of resting and foraging, whereas coarser sampling may only justify broader distinctions such as travelling versus encamped.

The choice of covariates should follow the same logic. Movement-only variables, such as SL and TA, are sufficient when the aim is to distinguish broad mobility modes, whereas environmental or directional covariates are needed when the goal is to identify context-dependent behaviours, such as orientation toward food patches, refuges, conspecifics, or other landscape features. In all cases, the proposed ordering rules should be grounded in established movement ecology theory and, whenever possible, in empirical evidence from the species or a closely related system. Existing studies can then be used to determine the expected qualitative pattern of each metric across states, for example whether SL, angular concentration, or directional persistence should increase or decrease from one behavioural state to another.

Accordingly, we define a small set of candidate behavioural states a priori and specify only the qualitative expectations that distinguish them. These expectations are not imposed as hard constraints during fitting; rather, they serve as ecological validation criteria for assessing whether the estimated state-dependent parameters are interpretable for the focal system. This makes the framework adaptable across species and sampling designs, while still requiring that the final state definitions be biologically defensible and consistent with the resolution of the data.

### 2.5 Decoding behavioural states in animal movement

#### 2.5.1 Pre-processing.

The purpose of this first step is to prepare case-control movement data suitable for HMM-SSF analysis. This involves generating case-control data by sampling control steps from a user-specified distribution (e.g., uniform or exponential family). Covariates are also extracted to characterise both case and control locations (Fig 1a).

**Fig 1:**
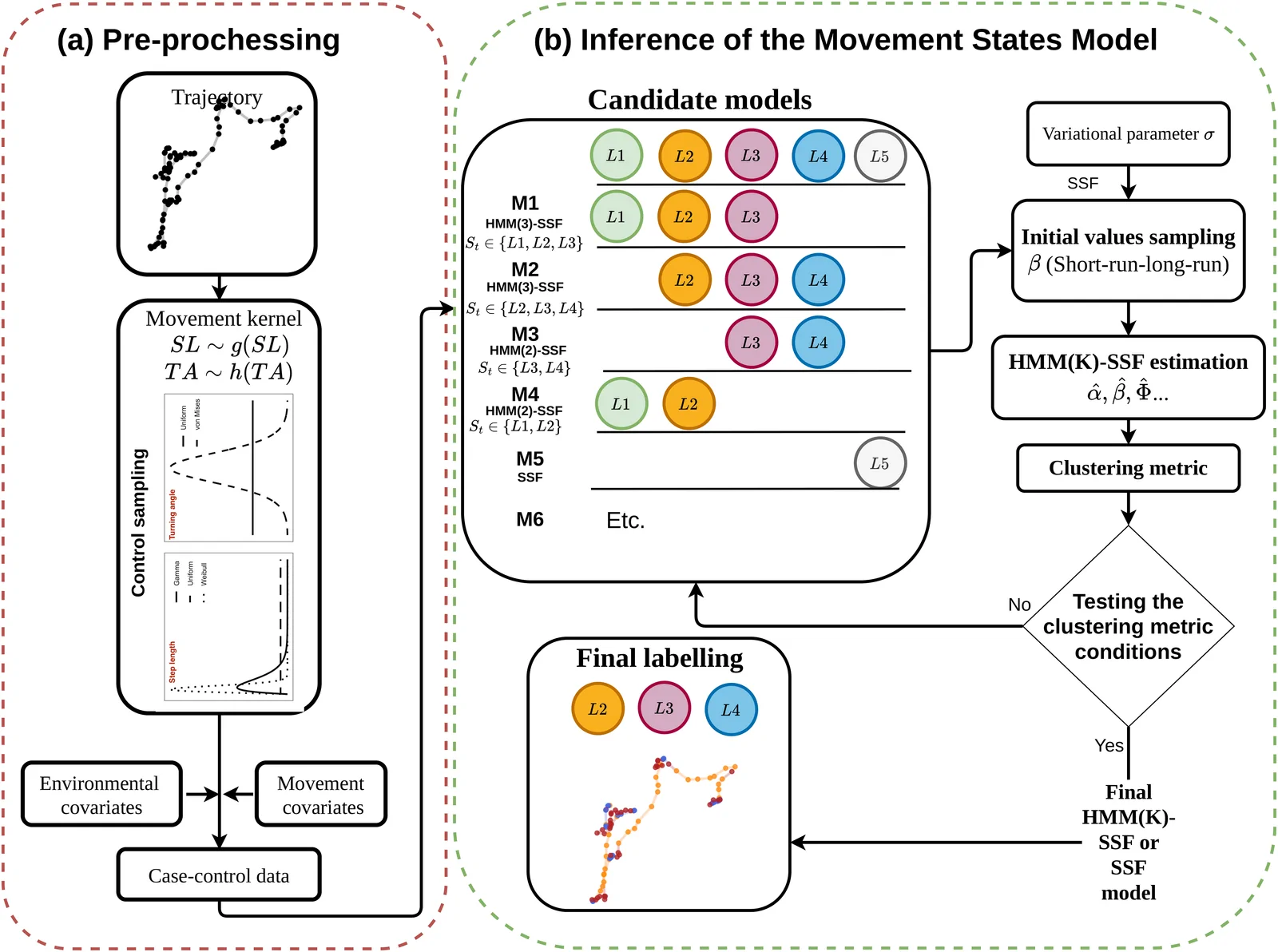
Inference framework for estimating the number of behavioural states and assigning ecological labels using the HMM-SSF. **(a)** Pre-processing: case-control data are prepared by sampling control steps and extracting environmental covariates. **(b)** Model fitting and state labelling: candidate HMM-SSF models are fitted using the short-run/long-run procedure, and states are labelled by evaluating whether estimated movement signatures satisfy predefined ordering and distinctness criteria. The most complex ecologically justified model is retained; simpler taxonomies are used as fallback.

#### 2.5.2 Short-run/long-run procedure.

The aim of the second step is to generate robust initial values for the β parameters to explore the plausible parameter space and reduce the risk of convergence to local likelihood maxima in the HMM-SSF likelihood surface [16]. A single-state SSF is then fitted via conditional logistic regression, which can be implemented using the clogit function from the amt package in R [34]. Based on the resulting estimates,*N* sets of initial parameter vectors are generated (where *N* is the number of random restarts), using:


$$\begin{array}{c} \beta_{i,j}^{\left(n\right)}\sim 𝒩({\hat{\beta_{i}}}^{j},\sigma_{i}^{2}),\;i=1,...,p,\;j=1,...,K,\;n=1,...,N \end{array}$$ (4)

where $\hat{\beta }$ denotes the mean estimate obtained from the conditional logistic regression, and $\sigma_{i}$ is a user-specified variational (spread) parameter controlling the dispersion of initial values around the single-state SSF estimate ${\hat{\beta }}_{i}$ for covariate *i*. We draw *N* candidate sets of initial values from the distributions in Eq. 4 $\{\beta^{\left(n\right)}{\}}_{n=1}^{N}$ for the HMM–SSF. For each candidate, we fit the full model with a short optimisation budget $n_{\text{iter\_short}}$ (the maximum number of iterations allowed in the short run) and record the maximised log-likelihood. We then select the best-performing run (largest log-likelihood) and restart the optimiser from its estimates using a longer number of iterations $n_{\text{iter\_long}}$ (the maximum number of iterations for the final long run). This “short-run/long-run” strategy [9] improves the selection of the starting values and reduces the risk of convergence to suboptimal local maxima.

#### 2.5.3 Calibration and testing.

The objective of this step (Fig 1b) is to determine the number of behavioural states and assign corresponding labels. We compile a list of candidate models that vary based on the study context corresponding to a priori hypotheses about the movement labels, and how these labels are converted into a single model.

**Model fitting** We fit the HMM-SSF model using direct likelihood maximisation via the forward algorithm [35] using the initial values from the short-run/long-run procedure. The likelihood of observing a sequence of locations $\left\{y_{1},...,y_{T}\right\}$ given the model parameters is expressed as:


$$\begin{array}{c} L\left(y_{1},...,y_{T}|\beta ,\Pi \right)=\delta \;Pr(y_{1},y_{2})\;\Pi \;Pr(y_{2},y_{3})\cdots \Pi \;Pr(y_{T-1},y_{T})\;1^{\prime } \end{array}$$ (5)

where $\delta =\left(Pr\left(S_{1}=1\right),...,Pr\left(S_{1}=K\right)\right)$ represents the initial distribution of hidden states (a row vector of length *K* whose *k*-th entry is the probability of starting in state *k*), $Pr\left(y_{t},y_{t+1}\right)$ is a diagonal matrix with the $j^{th}$ element representing the likelihood in Eq 2, and Π denotes the transition matrix of the Markov chain (the K×K matrix whose (*i*,*j*) entry is $\pi_{ij,t}$, the probability of moving from state *i* to state *j*). Maximum likelihood estimates of the HMM-SSF are obtained via direct likelihood maximization using the forward algorithm, implemented using the optim optimiser in the R package hmmSSF in R [17]. To estimate the hidden states, we can apply the Viterbi global decoding algorithm, which identifies the most probable sequence given the observations, i.e., the one that maximises $Pr\left(S_{1},...,S_{T}|y_{1},...,y_{T}\right)$ [35].

**Testing the order in the metrics** We begin by fitting a three-state HMM(3)-SSF incorporating the direction-to-target covariate, with $S_{t}\in \{HDT,\;F,\;R\}$. If Condition 1 is satisfied, in particular, if $\beta_{\cos(\theta -\theta^{\text{target}})}^{\left(HDT\right)}$ is statistically significant, we retain this model as the final result. If $\beta_{\cos(\theta -\theta^{\text{target}})}^{\left(HDT\right)}$ is not significant, we refit a three-state HMM(3)-SSF without the direction-to-target covariate, seeking $S_{t}\in \{T,\;R,\;F\}$ (see Condition 2). If the behavioural-state in Condition 2 are still not met, we fit the two-state HMM(2)-SSF with $S_{t}\in \{T,\;E\}$ (Condition 3). If no HMM with K≥2 satisfies the criteria, the model is simplified to a single-state SSF.

Candidate model space, and thus the set of behavioural labels, draws from five predefined modes {HDT, T, E, R, F}, the number of states can be defined by the user depending on the problem but we cap the number of estimated states at $K_{\max}=3$. Not all five modes can occur simultaneously: some are nested or mutually exclusive at the sampling scale (e.g., Resting vs. Encamped). Accordingly, admissible specifications are limited to biologically meaningful subsets.

### 2.6 Post-fit pruning and post-filtering rules

Our post-fit pruning and post-filtering procedure follows the same notation and logic as Algorithm 1. Starting from $K_{\max}=3$ with the direction-to-target covariate, we fit the HMM–SSF to obtain SSF parameters $\hat{\beta }$ and transition probability matrix $\hat{\Pi }$, decode the most likely state sequence ${\hat{S}}_{1:T}$ (Viterbi), and compute state occupancy proportions $p_{k}=T^{-1}\sum_{t=1}^{T}1\{{\hat{S}}_{t}=k\}$ (the fraction of time steps assigned to state *k* by the Viterbi decoder). We retain the first model that satisfies the ensemble of constraints described below; otherwise we reduce the model complexity as described in Algorithm 1.

1) **Numerical stability screening via TPM diagonals.** To avoid degenerate transition structures, we screen fits using the diagonal of the estimated TPM. Let $diag(\hat{\Pi })=\{{\hat{\pi }}_{11},...,{\hat{\pi }}_{KK}\}$ denote the vector of self-transition probabilities, where ${\hat{\pi }}_{kk}$ is the estimated probability of remaining in state *k* at the next time step. A fit is considered stable only if all self-transition probabilities lie within prescribed bounds,

$$\begin{array}{c} \max diag(\hat{\Pi })<\overset{―}{\pi }\;\text{and}\;\min diag(\hat{\Pi })>\underset{―}{\pi }, \end{array}$$

where $\left(\underset{―}{\pi },\overset{―}{\pi }\right)$ are user-specified lower and upper bounds on the self-transition probabilities. In our implementation, we use $\left(\underset{―}{\pi },\overset{―}{\pi })=(0.05,0.95\right)$. These thresholds are chosen to exclude degenerate transition regimes and can be adapted depending on temporal resolution.

2) **Occupancy-based pruning using Viterbi decoding.** We require that the Viterbi path ${\hat{S}}_{1:T}$ supports all fitted states and that each state has non-negligible occupancy. Specifically, we enforce: (i) $Card\left(\{{\hat{S}}_{t}{\}}_{t=1}^{T}\right)=K$ (all states are visited), and (ii) $\min_{k}p_{k}\geq \tau_{occ}$, where $\tau_{occ}$ is the minimum occupancy threshold (the smallest acceptable proportion of time steps that must be assigned to any given state). We set $\tau_{occ}=0.05$. If either condition fails, the model is refitted with K←K−1.
3) **Interpretability: ordering, distinctness, and label-specific constraints.** For each candidate *K* that passes the TPM and occupancy screens, we compute state-level metrics $\left\{\Psi_{k}\right\}$ from $\hat{\beta }$ (as in Algorithm 1) and attempt a label mapping based on movement summaries derived from $\hat{\beta }$ (e.g., ordered mean SL $\mu_{SL}$ and directional persistence proxies). The mapping is accepted only if the *label-specific constraints* encoded are satisfied, namely:
  - **Ordering constraints:** labels are assigned consistently along the ordered $\mu_{SL}$ gradient (larger $\mu_{SL}$ corresponding to faster displacement modes, smaller $\mu_{SL}$ to slower/resting modes).
  - **Distinctness constraints:** candidate labels must correspond to states that differ meaningfully in their estimated movement/SSF signatures (e.g., contrast in $\mu_{SL}$ and in key directional coefficients used by the code to separate targeted vs. non-targeted movement and foraging/resting-type states).

If the label mapping fails these constraints for a given *K*, we decrease *K* and repeat the fitting and filtering.

4) **Final selection and fallback.** The selected number of states $\hat{K}$ is the first (largest) *K* encountered in the descending search that satisfies the stability condition on $diag(\hat{\Pi })$, the occupancy constraint $\min_{k}p_{k}\geq \tau_{occ}$, and the label-specific constraints. If no multi-state solution satisfies these requirements, we fit a 1-state SSF as fallback, compute $\Psi_{1}$, and assign the label “Other”, returning $\left(1,\Psi_{1},\text{Other},{\hat{S}}_{1:T}\right)$.

**User responsibility.** Because behavioural labels remain scientific constructs, users must verify that the final output $\left(\hat{K},\{\Psi_{k}\},\text{labels},{\hat{S}}_{1:T}\right)$ is biologically coherent for the study system. In practice, we recommend inspecting the retained $\hat{\Pi }$, the occupancy proportions $\left\{p_{k}\right\}$, and the state-specific movement summaries implied by $\hat{\beta }$ (e.g., step-length and turning-angle patterns) to confirm that the selected labelling is consistent with domain knowledge.

### 2.7 Simulation study

Following the principles outlined in Section 2.3, we simulated the movement of computer agents across six scenarios (Table 2). Each scenario was designed to evaluate the framework’s ability to: (1) detect the true number of behavioural states, (2) accurately assign behavioural labels to those states, and (3) reliably reconstruct the sequence of behavioural states along movement trajectories over time. Scenarios 1–5 cover one- to three-state models under varying transition probabilities, while Scenario 6 extends the evaluation to a three-state model (HDT, Foraging, Resting) in which the habitat-directed travelling state incorporates a directional bias coefficient $\omega^{\left(HDT\right)}=5$, while Foraging and Resting states have ω=0. We also assessed the framework’s sensitivity to variations in state transition probabilities and the choice of sampling distribution for generating control locations based on the following distributions:

1. Case (GU_vMU): This corresponds to uniform sampling for controls. Movement is modelled using a gamma distribution for SL and a von Mises distribution for TA.
2. Case (GG_vMvM): In this case, both movement and control distributions are modelled using gamma-distributed SLs and von Mises-distributed TAs.

**Table 2:** Parameter values used to simulate movement paths. For Scenario 6, a state-specific directional bias coefficient ω is included to simulate habitat-driven movement in the HDT state (see Eq 7); all other states have ω=0. For the HDT state in Scenario 6, the angular concentration $\ell =\|V_{t}\|$ varies at each step (see Eq 7) and is therefore not a fixed parameter; the value reported for κ is the persistence component only.

Scenario	Labelling	State	$\mu_{SL}$	$\sigma_{SL}$	κ	ℓ	ω	*K*	Transition matrix Π
1	Other (Travelling)	O	15	5	10	10	0	1	–
2	Travelling, Encamped	T	15	5	10	10	0	2	0.8 0.2
		E	3	1	3	3	0		0.4 0.6
3	Travelling, Encamped	T	15	5	10	10	0	2	0.2 0.8
		E	3	1	3	3	0		0.4 0.6
4	Travelling, Encamped	T	15	5	10	10	0	2	0.8 0.2
		E	3	1	3	3	0		0.8 0.2
5	Travelling, Resting, Foraging	T	15	5	10	10	0	3	0.80 0.15 0.05
		R	0.3	0.1	0.5	0.5	0		0.10 0.70 0.20
		F	5	3	−5	5	0		0.20 0.10 0.70
6	HDT, Foraging, Resting	HDT	15	5	10	$\left\|V_{t}\right\|$	5	3	0.85 0.10 0.05
		F	5	3	−5	5	0		0.15 0.75 0.10
		R	0.3	0.1	0.5	0.5	0		0.05 0.20 0.75

For the uniform sampling, we compared the results for *J* = 20 and *J* = 200 to examine the effect of the weak law of large numbers on the convergence from HMM-SSF to HMM with GRW [16].

For each scenario, movement paths were generated using a Hidden Markov Model incorporating a correlated random walk, with


$$\begin{array}{c} \left\{ \begin{array}{c} TA_{t}|S_{t}=k\sim \text{vM}\left(\mu_{t}^{\left(k\right)},\ell^{\left(k\right)}\right)\\ SL_{t}|S_{t}=k\sim \text{Gamma}\left(\mu_{SL}^{\left(k\right)},\sigma_{SL}^{\left(k\right)}\right) \end{array}\right. \end{array}$$ (6)

Here, $\mu_{t}^{\left(S_{t}\right)}$ and $\ell^{\left(S_{t}\right)}$ denote the direction and angular concentration of the mean direction vector


$$\begin{array}{c} V_{t}=\kappa^{\left(S_{t}\right)}(\begin{array}{c} \cos\left(\theta_{t-1}\right)\\ \sin\left(\theta_{t-1}\right) \end{array})+\;\omega^{\left(S_{t}\right)}(\begin{array}{c} \cos\;\left(\theta_{t}^{target}\right)\\ \sin\;\left(\theta_{t}^{target}\right) \end{array}), \end{array}$$ (7)

where $\theta_{t-1}$ denotes directional persistence from the previous step and $\theta_{t}^{target}$ the bearing toward the nearest habitat target. The mean direction is $\mu_{t}=atan2(V_{t,y},\;V_{t,x})$ and the angular concentration is $\ell^{\left(S_{t}\right)}=\|V_{t}\|$. When $\omega^{\left(S_{t}\right)}=0$, this reduces to $\ell^{\left(S_{t}\right)}=|\kappa^{\left(S_{t}\right)}|$ (standard CRW). When $\omega^{\left(S_{t}\right)}\neq 0$, the concentration $\left\|V_{t}\right\|$ depends jointly on $\kappa^{\left(S_{t}\right)}$, $\omega^{\left(S_{t}\right)}$, and the angle between the persistence direction $\theta_{t-1}$ and the target bearing $\theta_{t}^{target}$, so the effective directional focus of movement increases when both terms reinforce each other. Setting $\omega^{\left(S_{t}\right)}=0$ for all states recovers the pure CRW used in Scenarios 1–5. For the HDT state in Scenario 6, $\omega^{\left(HDT\right)}=5$; all other states have $\omega^{\left(k\right)}=0$. The values of ω for each state and scenario are reported in Table 2. The target location was fixed at the origin, and $\theta_{t}^{target}$ was recomputed at each time step from the agent’s current position. $(S_{t}{)}_{t=1}^{T}$ was generated from a discrete-time first-order Markov chain with transition probability matrix Π and initial distribution δ; the initial state *S*_1_ was drawn from a uniform distribution.

In the simulation study, the maximum number of states was set to $K_{\max}=3$, reflecting the fact that no simulated scenario exceeds three behavioural states. We applied Condition 2 to Scenario 5 (Travelling–Resting–Foraging), Condition 3 to Scenarios 2–4 (Travelling–Encamped), and Condition 1 to Scenario 6 (HDT, Foraging, Resting).

## 3 Results

### 3.1 Framework verification: simulation study

#### 3.1.1 Number of states and control sampling distribution.

The simulated movement trajectories (Fig 2) exhibited the expected behavioural patterns: scenarios involving Travelling show larger ranges and longer SLs, whereas distinct TAs differentiate Travelling from Foraging behaviours.

**Fig 2:**
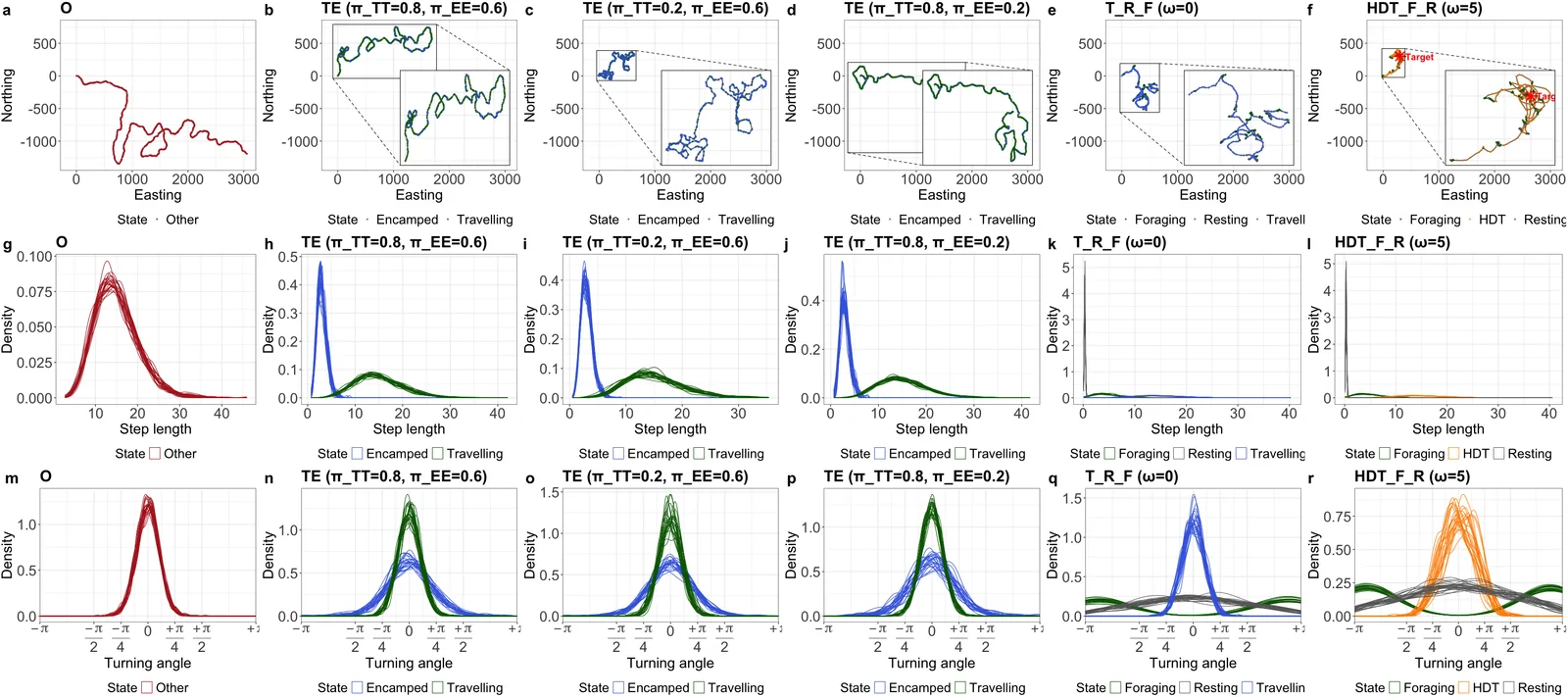
Simulated movement trajectories and state-dependent SL and TA distributions. Columns correspond to: Scenario 1 (O), Scenarios 2–4 (Travelling–Encamped under varying $\pi_{TT}$ and $\pi_{EE}$), Scenario 5 (TRF), and Scenario 6 (HDT/R/F, $\omega^{\left(HDT\right)}=5$). Rows show: (1) a representative trajectory coloured by true state, (2) SL distributions, and (3) TA distributions. Travelling states show longer SLs and more concentrated forward-directed TAs, while Encamped, Resting, and Foraging states show shorter SLs and more dispersed or reversed TAs. Each trajectory was simulated over 500 steps.

State-number identification was best under GG_vMvM (*J* = 20) sampling, which achieved 100% correct classification in all scenarios except Scenario 1, where 4% of individuals were assigned to *K* = 2 rather than the true $K^{*}=1$. Under GU_vMU (*J* = 200), performance remained high in most scenarios: all individuals were correctly identified in Scenarios 1 and 3, and 96% in Scenarios 2 and 4. However, 8% of individuals in Scenario 5 (TRF) were still assigned to a two-state model (*K* = 2) rather than the correct three-state model, and 4% of individuals in Scenario 6 (HDT/R/F) were reduced to a two-state solution. In contrast, GU_vMU (*J* = 20) produced the most errors across all scenarios. In Scenario 1 (O), all individuals were correctly assigned to *K* = 1. For the two-state scenarios, the error rate varied strongly with transition probabilities: 52% of individuals were correctly assigned in Scenario 2 ($\pi_{TT}=0.8$, $\pi_{EE}=0.6$), dropping to only 16% in Scenario 3 ($\pi_{TT}=0.2$, $\pi_{EE}=0.6$) and 28% in Scenario 4 ($\pi_{TT}=0.8$, $\pi_{EE}=0.2$). For the three-state scenarios, 28% of individuals were correctly classified in Scenario 5 (TRF) while 48% were correctly classified in Scenario 6 (HDT/R/F), with the remaining individuals assigned to a two-state model (48%) or a single-state model (4%). These results are summarised in Fig 3.

**Fig 3:**
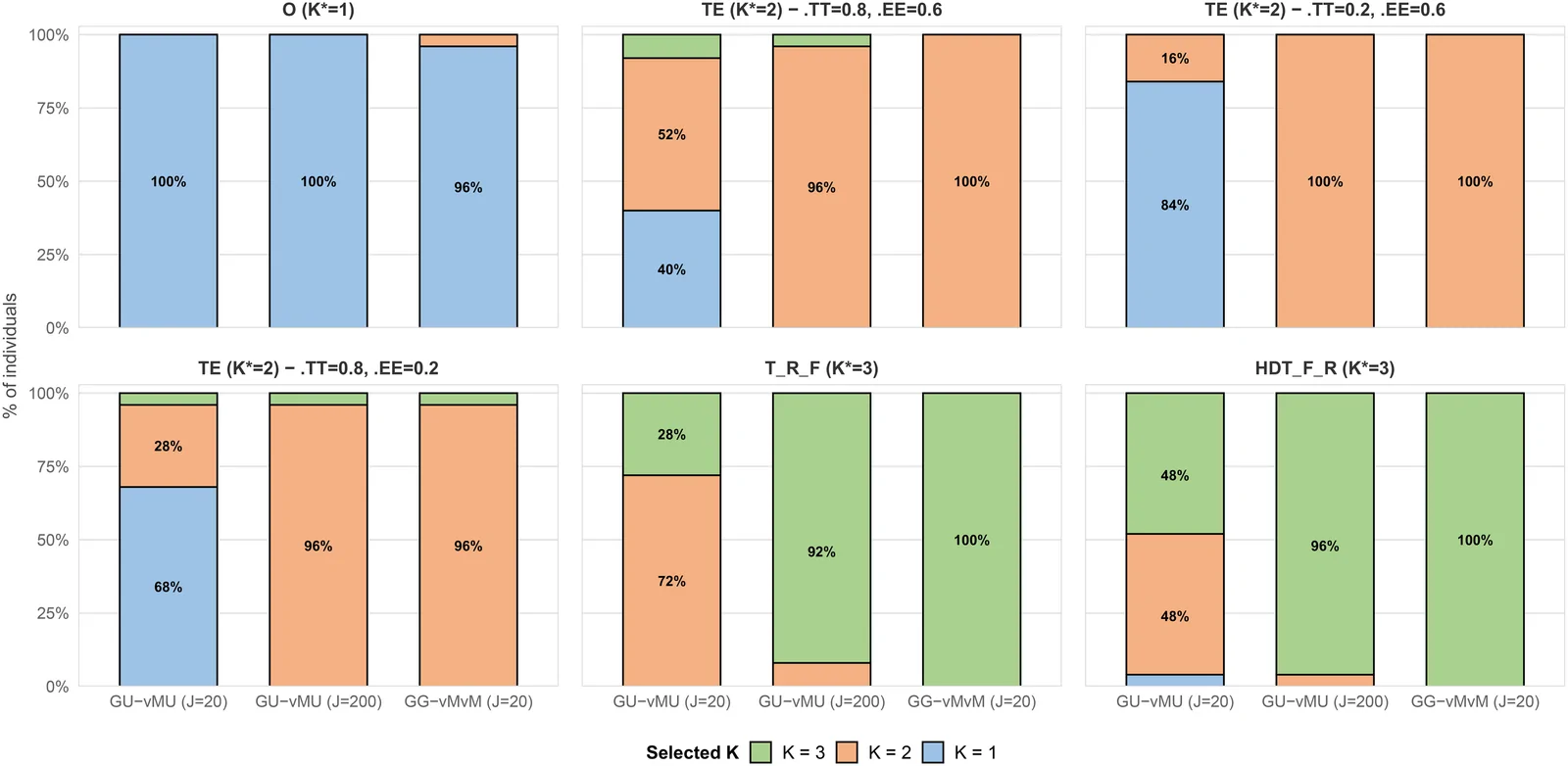
Effect of control sampling design on state-number selection. Each panel shows the proportion of individuals (*n* = 25) selecting *K* = 1 (blue), *K* = 2 (orange), or *K* = 3 (green) states under three control designs: GU-vMU (*J* = 20), GU-vMU (*J* = 200), and GG-vMvM (*J* = 20). Panels are arranged in two rows of three, one per scenario. True $K^{*}$ is shown in each panel title.

#### 3.1.2 State labelling and control distribution.

The identification of the correct state label (Fig 4) was least successful with uniform control point sampling with *J* = 20. For instance, in Scenario 4, the model incorrectly identified 17% and 23% of observations corresponding to travelling and encamped states. Similarly, in Scenario 3, encamped state labelling was only 5% accurate with uniform sampling at *J* = 20, compared to 99% accuracy with gamma and von Mises distributions (GG_vMvM (J = 20)). However, the performance of gamma and von Mises distributions for SL and TA remained highly accurate across all scenarios, with a maximum correct identification rate of 100% and a minimum of 93% for the traveling state in Scenario 5 (TRF) (Fig 4). With uniform sampling and *J* = 200 control points, performance under the three-state (TRF) model is slightly lower: the correct identification rates are 85% for foraging, 91% for resting and 95% for the traveling. Under the Gamma–von Mises control distributions, assignments are nearly perfect with only minor mislabeling within Scenario 5. A similar pattern holds for uniform sampling with *J* = 200, except in the three-state scenarios, where we observe noticeable mixing, primarily foraging as encamped and resting as encamped. For Scenario 6 (HDT/R/F), under GG_vMvM sampling the three states were perfectly discriminated with no mislabelling. Under both GU_vMU schemes, all three states were substantially mixed, suggesting that HDT and Resting/Foraging states are harder to distinguish from each other when using uniform control points.

**Fig 4:**
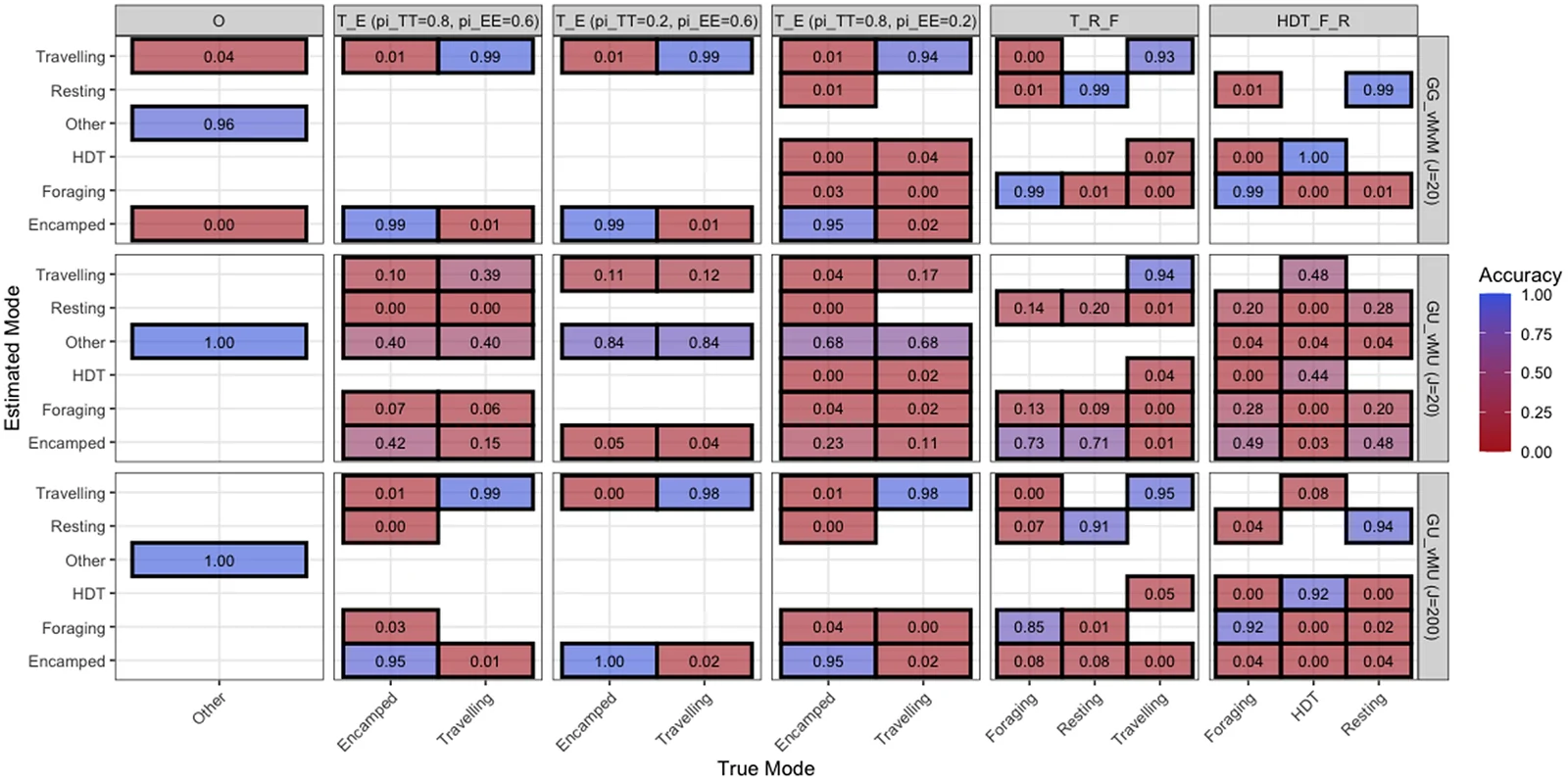
Accuracy of behavioural state classification. Overall classification accuracy of the estimated behavioural states relative to the true states across simulation scenarios and control sampling designs. Columns correspond to scenarios: O; TE with $(\pi_{TT},\pi_{EE})\in \{(0.8,0.6),\;(0.2,0.6),\;(0.8,0.2\}$; TRF; and HDT/R/F (with $\omega^{\left(HDT\right)}=5$). Rows correspond to control designs: GG_vMvM (*J* = 20), GU_vMU (*J* = 20), and GU_vMU (*J* = 200). Colour intensity reflects accuracy, ranging from red (low) to blue (high).

#### 3.1.3 State labelling and transition probability.

Reducing the probability of staying in the travelling state ($\pi_{TT}$) from 0.8 to 0.2 (Fig 4: Scenarios 2 and 3) had minimal effect on travelling state labelling under both GG_vMvM (J = 20) and GU_vMU (*J* = 200) control distributions. However, within the GU_vMU (*J* = 200) distribution, it led to a slight increase in the percentage of correctly identified encamped states from 95% to 100%.

#### 3.1.4 Sensitivity of HDT/F/R identification to directional bias strength and role of initial values.

The framework was applied to Scenario 6 (HDT/F/R) across four values of the directional bias coefficient $\omega^{\left(HDT\right)}\in \{1,5,8,10\}$, using GG_vMvM control sampling with *J* = 20. For each individual, the HMM(3)-SSF was fitted under Condition 1, with the initial value of $\beta_{\cos(\theta -\theta^{\text{target}})}^{\left(HDT\right)}$ set equal to the true ω value used in simulation (Fig 5).

**Fig 5:**
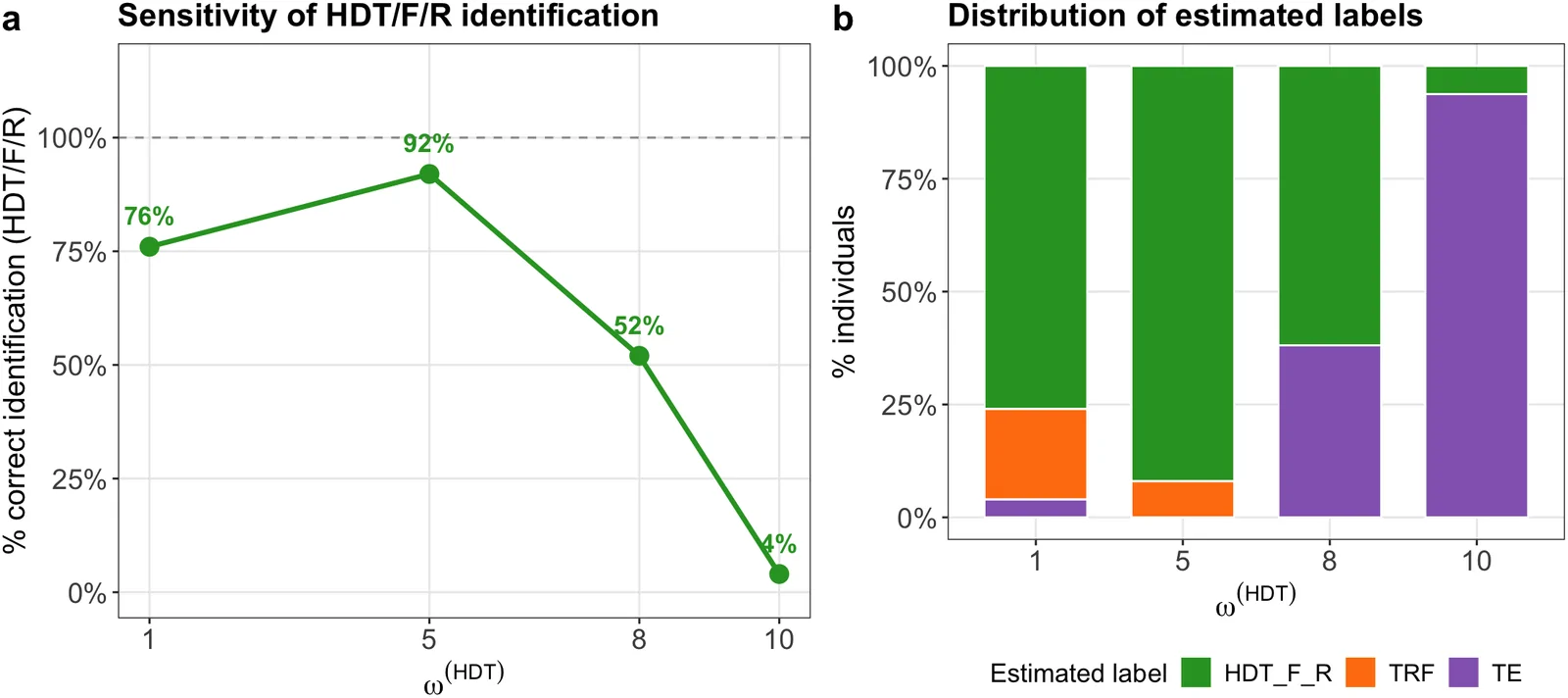
Sensitivity of HDT/F/R identification to directional bias strength $\omega^{\left(HDT\right)}$. **(a)** Percentage of individuals correctly identified as HDT/F/R as a function of $\omega^{\left(HDT\right)}$. **(b)** Stacked bar showing the distribution of assigned labels for each ω value: green = correct HDT/F/R; orange = T/R/F (target covariate non-significant); purple = T/E (collapsed to two states); grey = single state.

Correct identification of the HDT/F/R structure peaked at $\omega^{\left(HDT\right)}=5$ (92%), declined to 52% at ω=8, and collapsed to 4% at ω=10, where 92% of individuals were instead assigned to a two-state Travelling–Encamped model. Two distinct failure modes were observed. When ω was too small (ω=1), the model recovered the correct three-state structure but failed to detect a significant $\beta_{\cos(\theta -\theta^{\text{target}})}^{\left(HDT\right)}$, leading to downgrading to the T/R/F label (Condition 2; 20% of individuals). When ω was too large (ω≥8), the dominant failure was a collapse to the two-state T/E model, consistent with the increasing collinearity between $\cos(\theta -\theta_{t-1})$ and $\cos(\theta -\theta^{\text{target}})$ as the animal orients persistently toward the target, rendering the two angular covariates nearly indistinguishable.

#### 3.1.5 Comparison with HMM-BCRW state-number selection.

To contextualise the performance of the HMM-SSF framework, we compared its state-number selection against a standard hidden Markov model with biased correlated random walk (HMM-BCRW) fitted using the momentuHMM package [36], using the GG-vMvM (*J* = 20) control design. For each individual and each scenario, the HMM-BCRW was fitted with K∈{1,2,3} states and a circular-circular regression of the turning angle onto the direction toward the target. The best model was selected by AIC and, separately, by BIC. Results are shown in Fig 6.

**Fig 6:**
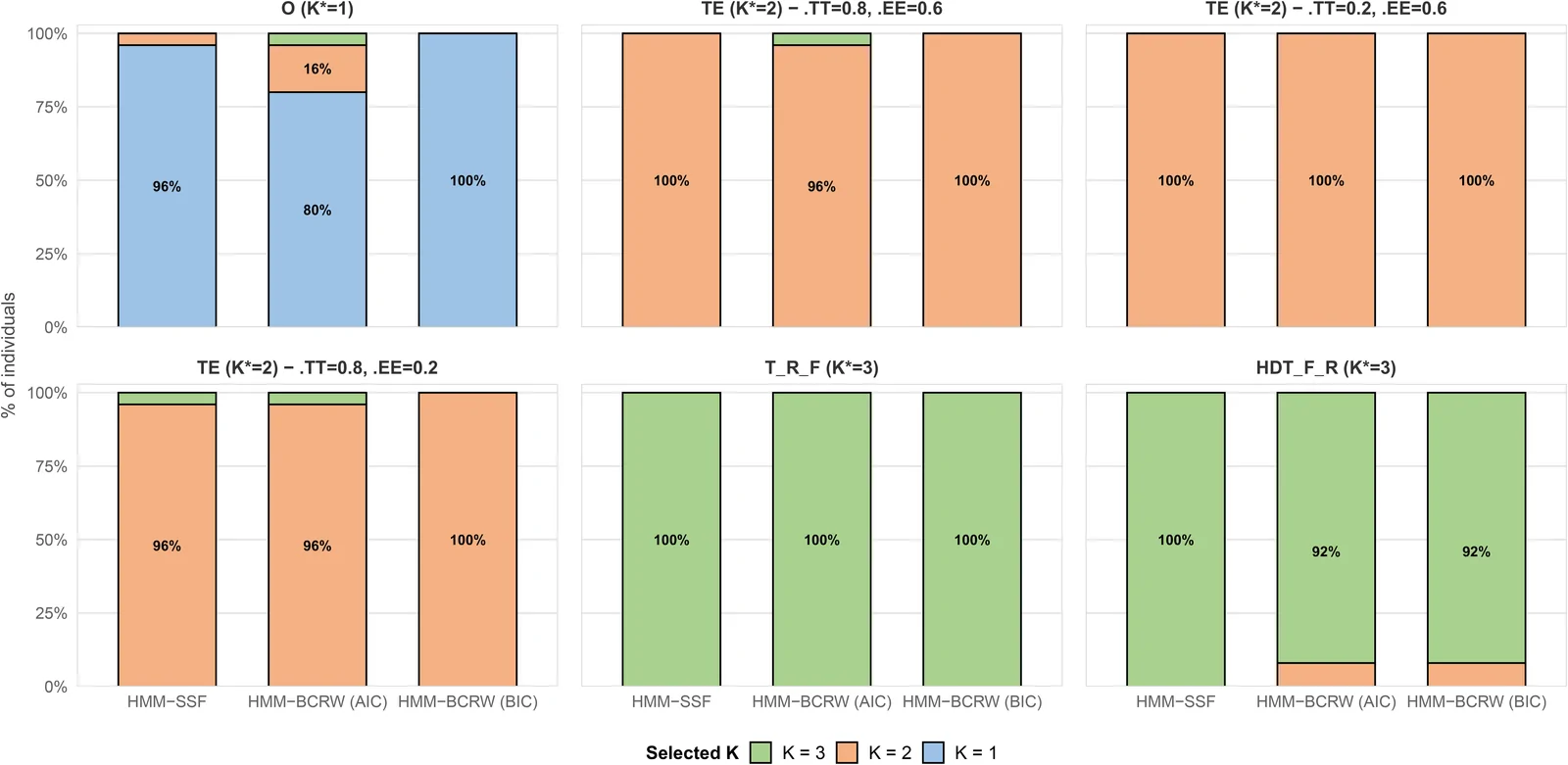
HMM-SSF vs HMM-BCRW state-number selection. Comparison of state-number selection between the HMM-SSF (ecologically guided, left bar per panel) and the HMM-BCRW using AIC (centre) or BIC (right), under the GG-vMvM (*J* = 20) control design. Each panel shows the percentage of individuals (*n* = 25) selecting *K* = 1 (blue), *K* = 2 (orange), or *K* = 3 (green) states. True $K^{*}$ is shown in each panel title.

Both methods selected the correct number of states in the majority of scenarios. For Scenario 1 (O, $K^{*}=1$), the HMM-SSF assigned 96% of individuals to *K* = 1, while HMM-BCRW (BIC) achieved 100% correct classification; however, HMM-BCRW (AIC) overestimated the state number for 20% of individuals (16% to *K* = 2 and 4% to *K* = 3). For the two-state scenarios (Scenarios 2–4), the HMM-SSF and HMM-BCRW (BIC) reached 100% correct classification. HMM-BCRW (AIC) overestimated the state number in Scenario 2, assigning 4% of individuals to *K* = 3 instead of the true $K^{*}=2$, and 4% in Scenario 4. For the three-state scenarios, all three methods achieved 100% correct classification in Scenario 5 (TRF). In Scenario 6 (HDT/R/F), the HMM-SSF correctly selected *K* = 3 for all individuals (100%), while both HMM-BCRW (AIC) and HMM-BCRW (BIC) selected *K* = 2 for 8% of individuals and *K* = 3 for the remaining 92%. Overall, the HMM-SSF achieved performance comparable to or better than HMM-BCRW (BIC) across all scenarios, and systematically outperformed HMM-BCRW (AIC) in scenarios where AIC overestimated the number of states. Importantly, beyond state-number selection, the HMM-SSF framework additionally provides biologically interpretable state labels grounded in explicit ecological ordering criteria, which the standard HMM-BCRW workflow does not.

### 3.2 Illustrative example

Here, we apply the framework presented in section 2.3 to an existing dataset consisting of GPS locations of plains zebras (*Equus quagga*) collected every 30 minutes between January and April 2014 (n = 7,246 locations from one individual) in Hwange National Park, Zimbabwe [17,37]. We added a habitat covariate, defined as the cosine of the difference between the animal’s movement direction and the direction toward the nearest grassland habitat. This covariate was included to test whether a habitat-directed travelling state could be detected.

We define the log-linear component of the state-specific likelihood in Eq 1 by


$$\begin{array}{c} c^{T}.\beta =\beta_{SL}SL+\beta_{log\_SL}\log(SL)+\beta_{\cos(TA)}\cos(TA)+\beta_{\cos(\theta -\theta^{\text{target}})}\cos(\theta -\theta^{\text{target}}) \end{array}$$ (8)

For the parameters used in the short-run/long-run procedure (Section 2.5.2), we considered a number of replications *N* = 10. The variation scale parameter (σ) was set to 1 for all parameters except for SL, where σ was set to 4. The number of iterations for the short run was set to $n_{\text{iter\_short}}=100$, and for the long run, it was set to $n_{\text{iter\_long}}=1000$.

The framework identified three behavioural states: Habitat-Dependent-Travelling (HDT), Foraging and Resting states. These states were well discriminated, as evidenced by smoothed state probabilities (Fig 7a) and the Viterbi-decoded state sequence (Fig 7b). The HDT state (blue line) represents directed movement toward the grassland habitat ($\beta_{\cos(\theta -\theta^{\text{target}})}>0$), whereas the Foraging and Resting states (green and red lines, Fig 7b) reflect localised behaviour. The HDT and Foraging and Resting states strongly differ in SL and TA distributions (Fig 7c and 7d):

- **HDT:** $\mu_{SL}^{\left(HDT\right)}=0.57$ km, $\kappa^{\left(HDT\right)}=2.66$, $\beta_{\cos(TA)}^{\left(HDT\right)}>0$ and significant $\beta_{\cos(\theta -\theta^{\text{target}})}^{\left(HDT\right)}>0$.
- **Foraging:** $\mu_{SL}^{\left(F\right)}=0.19$ km, $\kappa^{\left(F\right)}=1.61$, $\beta_{\cos(TA)}^{\left(F\right)}<0$ and significant $\beta_{\cos(\theta -\theta^{\text{target}})}^{\left(F\right)}>0$
- **Resting:** $\mu_{SL}^{\left(R\right)}=0.08$ km, $\kappa^{\left(R\right)}=0.34$, $\beta_{\cos(TA)}^{\left(R\right)}>0$ and $\beta_{\cos(\theta -\theta^{\text{target}})}^{\left(R\right)}>0$.

**Fig 7:**
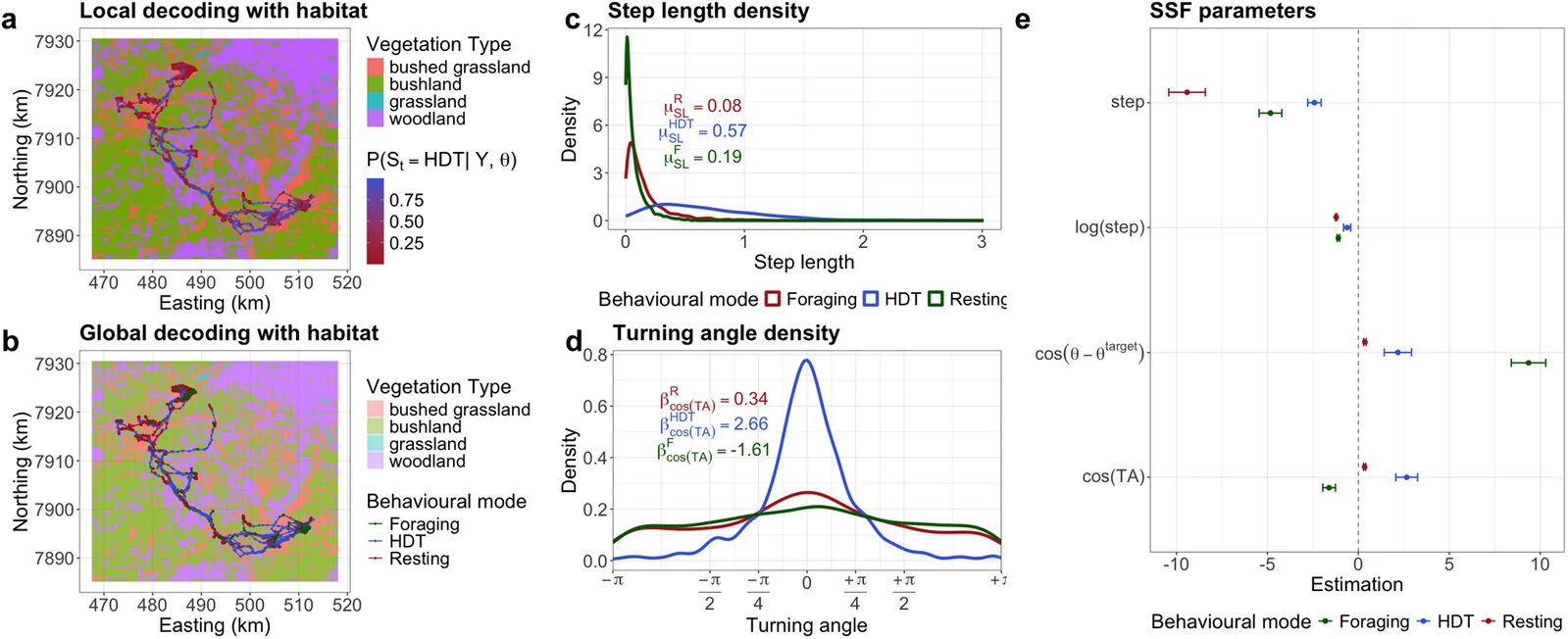
Application of the HMM-SSF framework to GPS tracking data from a plains zebra (*Equus quagga*). **(a)** Smoothed probability of the HDT state overlaid on vegetation types. **(b)** Viterbi-decoded state sequence. **(c)** SL distributions per state. **(d)** TA distributions per state. **(e)** State-specific vegetation selection coefficients with confidence intervals (see Tables A and B in S1 Appendix for estimation details).

We observed that the HDT state was meaningful; thus, in the three-state model (Condition 1), $\beta_{\cos(\theta -\theta^{\text{target}})}>0$ for both HDT and Foraging states. This indicates that animals were attracted toward grassland habitat in both modes.

The results differed from those reported in Klappstein et al. [17], where the two states considered were Travelling and Encamped. This discrepancy can be explained, first, by differences in the environmental variables included in the SSF: in our case, we incorporated the direction toward the preferred grassland habitat of zebra. Moreover, unlike their approach, which predefined the number of states, our method estimated both the number of states and their labels simultaneously. In Klappstein et al. [17], they also proposed a three-state model; our analysis empirically supports this three-state structure, as just demonstrated.

## 4 Discussion

Characterising behavioural states from animal movement data remains a central challenge in movement ecology, particularly in the absence of direct individual-level observations. We introduce a framework that integrates HMMs with step selection functions (SSFs) to infer both the number and interpretation of behavioural states characterizing the multiphasic structure of movement paths. The framework describes the order of selection in an unsupervised learning with ecological constraints by leveraging ecologically interpretable movement metrics, such as SL, angular concentration, and directional persistence, as clustering criteria. This design enables biologically grounded especially when the number or nature of states is uncertain. To balance model complexity and interpretability, the number of states is limited [38], as the number of possible models grows exponentially with each additional state (e.g., four models for three states, fourteen for four states; see Fig A in S1 Appendix).

Our framework also addresses practical challenges in applying SSFs to movement data, particularly the generation of control points, which can bias parameter estimates when poorly specified [25,39]. In our simulations, uniform control sampling with a small number of control locations (e.g., *J* = 20) often led to poor state differentiation, whereas increasing the number of controls to *J* = 200 or using parametric control distributions such as gamma for SL and von Mises for TA substantially improved inference. Notably, the parametric control design with *J* = 20 performed best overall, outperforming the uniform design even at *J* = 200, which suggests that matching the control distribution to the empirical movement process may be more important than simply increasing the number of controls. This improvement is consistent with the convergence of the SSF approximation to GRW behaviour as J→∞ [15,16]. However, if the goal is not to examine equivalence with the GRW formulation, the choice of *J* should be guided by the trade-off between computational cost and approximation accuracy rather than by asymptotic considerations alone. In addition, low persistence in some states, such as encamped behaviour, can lead to underestimation or mislabelling of states, which supports the use of semi-Markovian transition structures to better represent state duration and improve model realism [13,40].

In this study we specify a small set of candidate state taxonomies (e.g., Travelling–Encamped; Travelling–Resting–Foraging; and, habitat-dependent vs. habitat-independent travelling) and pair them with state-specific movement signatures and environmental drivers. Density-independent cues (orientation to landscape features and habitat structure) anchor displacement behaviours and cleanly separate habitat-dependent from habitat-independent travelling. Compared with information-criterion workflows that optimise fit but can yield behaviourally opaque states [21], our constrained, unsupervised strategy prioritises biologically defensible partitions while still estimating the latent sequence. This complements pragmatic order-selection tools by offering an ecologically anchored alternative when “best AIC/BIC” and “best biology” diverge [41,42]. It also accords with model-based clustering perspectives, in which “true” states are defined relative to study aims, temporal resolution, and covariate design rather than as unique, data-determined entities [43]. Finally, we determine the effective number of states via simple penalties on the transition structure and on state occupancy/labelling (minimum prevalence, persistence bounds, and refitting when empty states are detected), in contrast to approaches that rely on shrinkage or regularisation of state-specific coefficients [22]. A direct benchmark against the HMM-BCRW further contextualises these properties. The tendency of AIC to favour overly complex HMMs is well documented [21], and our simulations confirm this pattern: AIC selected more states than the true model in several scenarios, particularly those with low state persistence or a single true state. BIC, which imposes a stronger complexity penalty, recovered the correct state number more consistently, in close agreement with the HMM-SSF. This is broadly consistent with Pohle et al. [21]. The key distinction, however, is not merely which criterion performs better numerically: whereas both AIC- and BIC-based workflows treat order selection as a model-comparison problem to be solved by the data alone, the HMM-SSF framework embeds ecological constraints directly into the selection procedure, so that only models whose state-dependent signatures are biologically coherent are retained. This shifts the burden from post-hoc interpretation, where a researcher must decide after fitting whether a given *K*-state solution is meaningful, to pre-specification, where biological expectations are encoded before fitting and used to validate, rather than merely describe, the inferred state structure [43]. The result is a procedure that is robust to the known instability of information criteria in finite samples [21,22] while remaining grounded in the ecological question at hand.

The sensitivity analysis of the directional bias coefficient highlights two limitations that are not specific to our framework but reflect broader challenges in movement ecology. When directional bias is weak, the habitat-orientation covariate contributes too little to the likelihood surface to be reliably distinguished from background correlated movement, a problem common to habitat selection analyses more generally [25,39]. When bias is very strong, persistence and target orientation become nearly collinear, a well-recognised structural challenge in biased correlated random walk models [15,16] that limits the ability of any model to disentangle the two sources of directional information. These findings illustrate the risk of anchoring the initial value of the habitat-orientation coefficient to a strong prior estimate of bias magnitude: even when the true value is known, initialising near an extreme of the parameter space can impair convergence and inflate uncertainty. The framework is therefore most reliable when habitat-directed movement is a recognisable but not overwhelming component of the animal’s locomotion, and we recommend conservative initialisation of this coefficient rather than relying on prior expectations of its magnitude.

In summary, the suggested framework advances behavioural state inference from animal tracking data by integrating ecological theory, formal equivalence with movement models, and practical aspects such as control sampling and state persistence. Its combination of flexibility, interpretability, and statistical rigour makes it a powerful and generalisable tool for movement ecology.

## Supporting information

S1 AppendixModel selection tree and HMM-SSF estimates for the plains zebra.Fig A: General structure of the HMM-SSF framework for $K_{max}=4$. Table A: State-specific HMM-SSF coefficients with 95% confidence intervals. Table B: Estimated transition probability matrix.(PDF)
