# Supplementary material for: Behavioural state inference from movement and environmental data using Markovian step selection functions
Source: PLoS One. 2026 Oct 26;21(10):e0360547. doi: 10.1371/journal.pone.0360547 (PMC13705615; doi:10.1371/journal.pone.0360547)
Supplement: Supplementary file 1 [file pone.0360547.s001.pdf]

S1 Appendix. Model selection tree and HMM-SSF estimates for the plains zebra

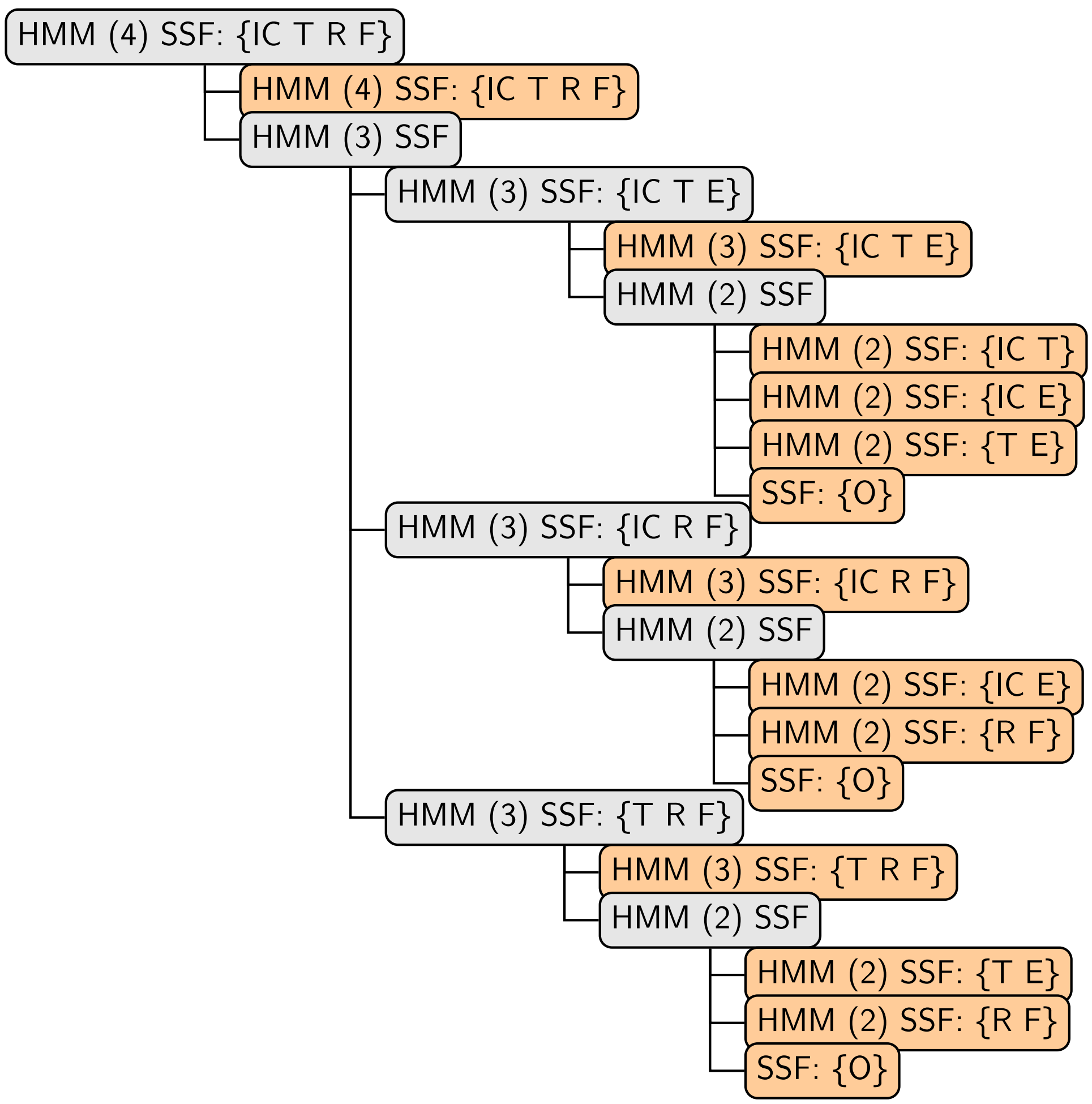

Fig A: **General structure of the HMM-SSF framework for $K_{max} = 4$.** Behavioural labels: inter-camp (IC), travelling (T), encamped (E), resting (R), foraging (F) and other (O). Grey rectangles represent the tested models, while orange rectangles indicate potential final models.

Table A: **State-specific HMM-SSF coefficients for the plains zebra.** Model: `~ step + log_step + cos_angle + cos_diff_theta_to_target`. Estimate: maximum likelihood estimate; Lower and Upper: bounds of the 95% confidence interval. HDT: habitat-dependent travelling; F: foraging; R: resting. Log-likelihood = −13 283.76.

Parameter	Estimate	Lower 95% CI	Upper 95% CI
SL (HDT)	−2.416	−2.783	−2.050
$\log(SL)$ (HDT)	−0.618	−0.814	−0.421
$\cos(TA)$ (HDT)	2.659	2.060	3.257
$\cos(\theta - \theta^{target})$ (HDT)	2.172	1.422	2.921
SL (F)	−4.832	−5.454	−4.210
$\log(SL)$ (F)	−1.104	−1.169	−1.038
$\cos(TA)$ (F)	−1.610	−1.960	−1.261
$\cos(\theta - \theta^{target})$ (F)	9.357	8.408	10.306
SL (R)	−9.417	−10.424	−8.411
$\log(SL)$ (R)	−1.223	−1.261	−1.185
$\cos(TA)$ (R)	0.343	0.283	0.403
$\cos(\theta - \theta^{target})$ (R)	0.359	0.292	0.427

Table B: **Estimated transition probability matrix (TPM) for the plains zebra.** Rows give the current state and columns the next state. HDT: habitat-dependent travelling; F: foraging; R: resting.

	HDT	F	R
HDT	0.605	0.136	0.260
F	0.103	0.806	0.091
R	0.049	0.096	0.854
